# Chronic Spontaneous Urticaria and Psychiatric Symptoms: The Blood–Brain Barrier Hypothesis

**DOI:** 10.1111/exd.70369

**Published:** 2026-09-29

**Authors:** Gerasimos N. Konstantinou, Jerry J. Warsh, Daniel M. Blumberger, Stefan Kloiber, George N. Konstantinou

**Affiliations:** ^1^ Department of Psychiatry University of Toronto Toronto Ontario Canada; ^2^ Centre for Addiction and Mental Health (CAMH) Toronto Ontario Canada; ^3^ Temerty Centre for Therapeutic Brain Intervention CAMH Toronto Ontario Canada; ^4^ Centre for Mental Health University Health Network (UHN) Toronto Ontario Canada; ^5^ Department of Allergy and Clinical Immunology 424 General Military Training Hospital Thessaloniki Greece; ^6^ Elliot and Roslyn Jaffe Food Allergy Institute, Serena and John Liew Division of Pediatric Allergy and Immunology, Jack and Lucy Clark Department of Pediatrics Kravis Children's Hospital, Icahn School of Medicine at Mount Sinai New York New York USA

**Keywords:** blood–brain barrier, chronic spontaneous urticaria, mast cells, neuroinflammation, substance P

## Abstract

Chronic spontaneous urticaria (CSU) is a mast cell‐driven inflammatory disease frequently accompanied by anxiety, depression, sleep disturbance, fatigue, and impaired quality of life. Pruritus, disturbed sleep, psychosocial burden, and treatment‐related effects are established contributors to this neuropsychiatric and functional burden. Whether immune‐to‐brain signalling provides an additional mechanism in a susceptible subgroup remains unknown. This hypothesis‐driven narrative review examines the blood–brain barrier (BBB) as one potential interface linking CSU‐associated inflammation to central effects. CSU studies report systemic inflammatory, vascular, and neuroimmune signals, including cytokine signatures, circulating matrix metalloproteinase‐9, and substance P–MRGPRX2‐related findings. Their potential neurovascular relevance is inferred from experimental or other disease settings rather than demonstrated in CSU per se. Our targeted search identified no human CSU studies assessing regional BBB permeability with dynamic contrast‐enhanced magnetic resonance imaging or tracer methods, blood–cerebrospinal fluid barrier function with the albumin quotient, serum S100B, or brain endothelial tight‐junction integrity, including claudin‐5 or occludin. Structural and functional neuroimaging findings in CSU do not establish barrier dysfunction. Systemic mastocytosis provides comparative evidence for mast‐cell‐related neuropsychiatric manifestations, although barrier involvement remains unproven. We therefore propose neurovascular alteration as a testable, non‐exclusive risk modifier whose relevance to CSU pathophysiology and psychiatric symptoms remains to be established.

**Abbreviations**
BBB: blood–brain barrier
BMI: body mass index
CNS: central nervous system
CRP: C‐reactive protein
CSF: cerebrospinal fluid
CSU: chronic spontaneous urticaria
DCE‐MRI: dynamic contrast‐enhanced magnetic resonance imaging
FcεRI: high‐affinity IgE receptor
H1: histamine H1
IDO: indoleamine 2,3‐dioxygenase
IgE: immunoglobulin E
IgG: immunoglobulin G
IL: interleukin
MMP‐9: matrix metalloproteinase‐9
MRGPRX2: Mas‐related G protein‐coupled receptor X2
MRI: magnetic resonance imaging
QAlb: CSF/serum albumin quotient
S100B: S100 calcium‐binding protein B
SP: substance P
TNF‐α: tumour necrosis factor‐α
UAS7: Urticaria Activity Score over 7 days
UCT: Urticaria Control Test

## 1 Introduction

Chronic spontaneous urticaria (CSU) is a mast cell‐driven inflammatory skin disease characterized by spontaneous, recurrent wheals, angioedema, or both for more than 6 weeks [1, 2, 3]. Chronic urticaria affects approximately 0.7% of the general population worldwide, with regional variation, and CSU represents the predominant chronic urticaria subtype in most clinical settings [2, 4]. Management follows a stepwise approach beginning with a standard‐dose second‐generation H1‐antihistamine, with updosing to as much as fourfold when required. In patients who remain inadequately controlled, international guidelines include add‐on omalizumab, dupilumab, or remibrutinib, with further treatment individualized according to clinical response and patient‐related factors [1]. Beyond visible skin signs, CSU has a substantial multidimensional burden: pruritus, pain, fatigue, sleep disruption, emotional distress, stigma, and impaired social and occupational functioning are prominent, particularly when disease control is poor [2, 5, 6, 7, 8].

Psychiatric symptoms and comorbid disorders are frequently reported in CSU. In a meta‐analysis of observational studies, chronic urticaria was associated with increased odds of anxiety symptoms (pooled OR 3.99) and depressive symptoms (pooled OR 2.94) versus controls, with CSU subgroup estimates of OR 6.62 and OR 6.13, respectively [9]. Across cross‐sectional studies, registry cohorts, and meta‐analyses, individuals with CSU/chronic urticaria report higher rates of depression, anxiety, and emotional distress than controls [9, 10, 11, 12, 13]. A large real‐world matched‐cohort study reported increased all‐cause mortality and a higher risk of a composite outcome of suicidal ideation or suicide attempts in CSU [13]. Importantly, a 2026 matched cohort restricted to adults without a prior psychiatric diagnosis addressed a different question, new‐onset psychiatric disease after CSU diagnosis, and found only a modest increase in incident stress and adjustment disorders, with no consistent increase in incident depression, schizophrenia, or suicide‐related outcomes over 3 years [14]. Thus, the substantial psychiatric burden observed in cross‐sectional and prevalence‐based analyses should not be interpreted as evidence that CSU broadly causes new psychiatric disease. Rather, the available data support a distinction between prevalent comorbidity and incident risk [1, 2, 10, 13, 14].

An important proportion of the psychiatric symptom burden in CSU may therefore arise through well‐established clinical consequences of the disease, particularly persistent pruritus and sleep disturbance, together with fatigue and psychosocial burden [7, 8]. In an adolescent mediation analysis, itching and sleep disturbance accounted for 65.4% and 77.6% of the associations between CSU and anxiety and depression, respectively, and the direct association was no longer significant after these mediators were modelled [15]. Because this was a cross‐sectional analysis in adolescents, it does not establish causality or exclude other mechanisms. Nevertheless, it supports symptom‐related pathways as major contributors while leaving open the possibility that additional biological processes may influence psychiatric risk in some patients.

One potential additional mechanism involves neurovascular signalling at the blood–brain barrier (BBB). The BBB is a dynamic interface that regulates immune‐to‐brain communication and can be influenced by systemic inflammation. However, direct evidence of BBB dysfunction in CSU is currently lacking. We therefore consider BBB/neurovascular modulation as a testable, non‐exclusive mechanism that could contribute to psychiatric risk in a subset of patients, alongside the established symptom‐related pathways described above [16, 17, 18, 19]. Figure 1 summarizes the current evidence and the proposed framework.

**FIGURE 1:**
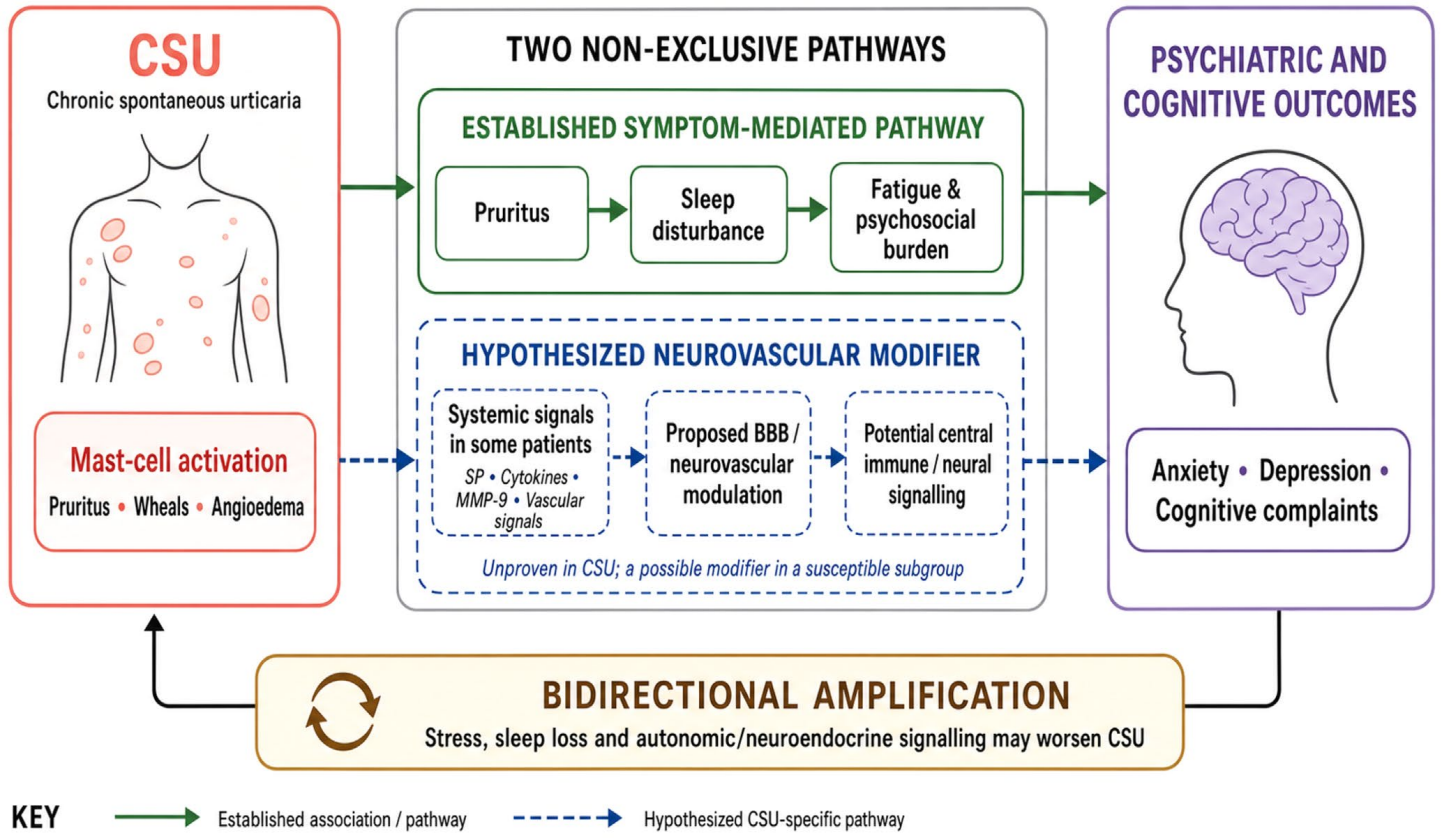
Evidence‐calibrated framework linking CSU, proposed neurovascular alterations, and psychiatric symptoms. Chronic spontaneous urticaria (CSU) is associated with psychiatric symptoms through an established symptom‐related pathway (pruritus, sleep disturbance, fatigue, and psychosocial burden) and, potentially, through an additional neurovascular pathway. Solid green arrows indicate established clinical associations; dashed blue arrows indicate hypothesized CSU‐specific steps. The proposed blood–brain barrier (BBB)/neurovascular pathway has not been demonstrated directly in CSU and is presented as a possible modifier in a susceptible subgroup. The lower feedback arrow denotes bidirectional amplification through stress, sleep loss, and autonomic/neuroendocrine signalling. MMP‐9, matrix metalloproteinase‐9; SP, substance P.

## 2 Scope and Literature Approach

This hypothesis‐driven narrative review was informed by targeted literature searches rather than a formal scoping‐review methodology. PubMed/MEDLINE, Web of Science, and Scopus were searched from database inception through 1 August 2026 using combinations of terms related to chronic spontaneous urticaria/chronic urticaria, psychiatric outcomes, pruritus, sleep disturbance, blood–brain barrier and neurovascular function, neuroinflammation, mast cells, substance P, MRGPRX2, cytokines, endothelial and coagulation markers, tight‐junction proteins, and systemic mastocytosis. We also examined reference lists of relevant reviews and primary studies to identify additional pertinent publications.

We prioritized peer‐reviewed human studies, meta‐analyses, systematic reviews, and clinical guidelines addressing psychiatric burden and established symptom‐related pathways in CSU, followed by CSU‐specific neuroimmune, vascular, and neuroimaging findings. We used evidence from systemic mastocytosis and psychiatric or other inflammatory disorders for comparison and included preclinical studies to define potential biological pathways. Throughout the review, we distinguished direct CSU evidence from comparative evidence and mechanistic extrapolation. Because the purpose was hypothesis generation rather than exhaustive evidence mapping, we did not use a protocol, duplicate screening, a formal risk‐of‐bias assessment, or a PRISMA‐ScR flow diagram.

## 3 Psychiatric Symptoms and Comorbidity in CSU: Established Burden

Multiple meta‐analyses and observational cohorts support a substantial burden of psychiatric symptoms and comorbidity in CSU/chronic urticaria, particularly anxiety, depression, stress‐related symptoms, and sleep disturbance [9, 10, 11, 12, 13, 14, 20]. This burden, however, should not be equated with a uniform increase in incident psychiatric disease after CSU diagnosis [14]. Although case definitions and psychiatric measures vary, sleep impairment is consistently common and is both a likely contributor to mood symptoms and a clinically actionable target [5, 8, 15]. Children and adolescents with chronic urticaria also show increased psychiatric comorbidity, indicating that this burden is not limited to adults [21].

These findings have direct clinical implications: clinicians managing CSU should routinely assess sleep disturbance and common depressive and anxiety symptoms, evaluate for suicidal ideation when clinically indicated, and pursue complete disease control using guideline‐recommended therapy [1, 2, 6, 13, 22]. The proposed BBB framework does not currently justify changes to clinical management: BBB dysfunction has not been demonstrated in CSU, and no BBB‐directed intervention can be recommended on this basis. Its present value is to generate stratified, testable research questions.

## 4 The BBB in Immune‐To‐Brain Communication

The BBB forms primarily from specialized brain microvascular endothelial cells joined by tight junctions and supported by pericytes, astrocytic endfeet, the basement membrane, and other components of the neurovascular unit [16, 17]. It is not merely a passive barrier, but a dynamic interface that regulates molecular transport, immune‐cell trafficking, and communication between the systemic circulation and the central nervous system. Systemic inflammatory mediators, including circulating cytokines, can alter endothelial function, tight‐junction organization, transporter activity, and vesicular transport, and under some conditions increase barrier permeability and promote neuroinflammatory responses [18, 19]. Importantly, these changes may be subtle, transient, or regionally restricted and may affect BBB function even without overt barrier leakage.

Peripheral inflammation can also influence the brain without requiring increased BBB paracellular permeability. Immune‐to‐brain communication may occur through endothelial signalling, neural pathways including vagal afferents, circumventricular regions with specialized vasculature, and the blood–CSF interface at the choroid plexus [18, 19, 23, 24]. Pro‐inflammatory cytokines can engage these pathways and activate central autonomic, neuroendocrine, and immune responses, thereby affecting behaviour and mood without necessarily causing structural disruption of the BBB [18, 19, 23, 24]. These mechanisms are relevant to CSU because they provide biologically plausible routes by which systemic inflammatory and stress‐related signals could influence central nervous system function, even without overt BBB permeability changes.

Accordingly, immune‐to‐brain communication should not be equated with frank BBB breakdown. Functional alterations in endothelial signalling, transport, or neurovascular regulation may occur without measurable increases in barrier permeability, whereas overt leakage represents only one manifestation of neurovascular dysfunction [18, 19].

## 5 CSU‐Specific Neurovascular Evidence: What Has and Has Not Been Measured

Our targeted search did not identify human CSU studies assessing regional BBB permeability with DCE‐MRI or tracer methods, blood–CSF barrier function or CSF dynamics with QAlb or CSF total protein, serum S100B, or brain endothelial tight‐junction integrity (including claudin‐5 or occludin). These measures interrogate different biological compartments and should not be grouped as interchangeable “BBB biomarkers”. QAlb primarily reflects blood–CSF barrier function and CSF dynamics. Serum S100B is nonspecific and can be influenced by astroglial activation and extracranial sources. DCE‐MRI estimates regional BBB permeability [25, 26, 27, 28, 29].

Barrier‐related research in CSU has instead focused mainly on intestinal epithelial permeability. Five serum zonulin studies have produced divergent findings: one found no significant case–control difference but reported an association with UAS7, another found lower levels in CSU, two reported higher levels, with inconsistent relationships to angioedema, concomitant inducible urticaria, or disease activity, and a recent haptoglobin study found no case–control difference in zonulin but an association with poorer disease control [30, 31, 32, 33, 34].

CSU‐specific data provide stronger support for peripheral vascular and endothelial involvement. A systematic review identified endothelial activation, coagulation/fibrinolysis abnormalities, and circulating matrix metalloproteinase‐9 (MMP‐9) among the vascular signals reported in chronic urticaria [35]. Individual MMP‐9 studies are inconsistent: one reported an association with disease severity, another found elevated MMP‐9 that was neither disease‐specific nor correlated with urticaria activity, and a later study described changes during omalizumab treatment [36, 37, 38]. These findings justify investigation of neurovascular interfaces, but the markers are nonspecific and cannot serve as surrogate evidence of BBB dysfunction.

Brain‐imaging studies provide a separate line of CSU‐specific evidence. Structural and resting‐state functional MRI studies have reported alterations involving the striatum, cerebellum and reward–sensorimotor networks, premotor and fusiform regions, thalamocerebellar circuits, and orbitofrontal–striatal or orbitofrontal–hippocampal connectivity [39, 40, 41, 42, 43]. Several findings correlated with UAS7, pruritus, immunoglobulin E (IgE), or anxiety, and one longitudinal study reported functional changes alongside clinical improvement [39, 40, 41, 42, 43]. These studies provide objective evidence of structural or functional brain differences in CSU, although some reports may involve overlapping cohorts. None assessed regional BBB permeability, blood–CSF barrier function, or neuroinflammation. Therefore, they do not establish barrier dysfunction.

## 6 BBB‐Related Findings in Psychiatric Disorders: Implications for CSU Research

Systematic reviews and meta‐analyses suggest that brain‐barrier‐related measures may be altered in subsets of severe mental disorders, but the findings are heterogeneous and rely largely on indirect biomarkers. A meta‐analysis found higher circulating S100B in schizophrenia‐spectrum disorders, major depression, and bipolar disorder; however, between‐study heterogeneity was substantial, relevant confounders were often not accounted for, and S100B has extracranial sources [26]. A separate meta‐analysis reported higher CSF total protein and QAlb in schizophrenia‐spectrum and affective disorders [25]. These latter indices are more appropriately interpreted as possible alterations in blood–CSF barrier function and/or CSF dynamics than as direct evidence of brain endothelial BBB leakage [25, 26]. Together, these findings support general biological plausibility but do not establish a disorder‐specific barrier signature or causality [44].

A systematic review of 17 studies also reported altered serum claudin‐5 and occludin levels in several psychiatric disorders, with findings varying by protein and diagnosis [45]. These peripheral measurements should not be equated with direct evidence of brain endothelial tight‐junction disruption or increased regional BBB permeability [16, 17, 45].

Methodological lessons from psychiatric brain‐barrier research are relevant to CSU. Reported abnormalities appear subgroup‐specific, making stratification by inflammatory phenotype, metabolic comorbidity, and illness severity essential [25, 26]. The choice of readout also matters: DCE‐MRI, QAlb/CSF total protein, and circulating S100B interrogate different compartments or biological processes and cannot be used interchangeably. Factors such as BMI, smoking, cardiovascular disease, psychotropic medication, and acute infection or inflammation may also influence these measures and should be recorded and controlled [25, 26]. Applying these principles to CSU calls for well‐phenotyped cohorts, validated measures of disease activity, control, sleep, and psychiatric symptoms, and compartment‐appropriate neurovascular assessments.

## 7 CSU Biology That May Intersect Neurovascular Regulation

CSU is biologically heterogeneous. Alongside Type I autoallergic and Type IIb autoimmune endotypes, non‐IgE‐dependent mast‐cell activation, neuroimmune interactions, coagulation and complement activation, and persistent low‐grade inflammation may contribute in different patient subsets [2, 46, 47]. Circulating CRP, cytokines, coagulation factors, and other inflammatory markers can reflect disease activity or treatment response, although they do not specify a single neurovascular phenotype or disorder [35, 46, 47]. Systemic inflammatory signalling is relevant to the present hypothesis because cytokines and vascular mediators have been shown to influence brain endothelium and immune‐to‐brain communication in other inflammatory settings [18, 19].

Several CSU‐associated signals are therefore candidates for neurovascular modulation, but their BBB relevance is derived largely from non‐CSU experimental or inflammatory models. Table 1 summarizes the direct CSU evidence, potential neurovascular relevance, and principal limitation for cytokines, coagulation/endothelial activation, MMP‐9, substance P (SP), and MRGPRX2‐mediated mast‐cell activation [18, 19, 35, 36, 37, 38, 39, 40, 41, 42, 43, 46, 47, 48, 49, 50, 51, 52, 53, 54, 55, 56, 57].

**TABLE 1:** Candidate CSU signals with potential BBB/CNS relevance.

Signal/pathway	CSU‐specific evidence	Potential neurovascular/CNS relevance and limitation	Supporting evidence (ref #)
IL‐6/TNF/acute‐phase response	Elevated CRP and inflammatory cytokine signatures occur in subsets of CSU and may track with activity.	Systemic cytokines can activate brain endothelium in other inflammatory settings; no direct BBB measure has linked these signals to CNS outcomes in CSU.	[18, 19, 46, 47]
Coagulation and endothelial activation	D‐dimer, prothrombin fragment 1 + 2, adhesion molecules, tissue factor, P‐selectin, and related markers support peripheral vascular involvement.	These are peripheral vascular/endothelial signals, not BBB‐specific biomarkers.	[35, 46, 47]
MMP‐9	MMP‐9 is elevated in some chronic urticaria cohorts, but associations with activity and treatment response are inconsistent.	MMP‐9 can affect extracellular matrix and tight‐junction proteins in other contexts; circulating MMP‐9 does not demonstrate BBB dysfunction in CSU.	[35, 36, 37, 38, 48]
Substance P	Serum SP is increased in some cohorts and associated with activity or comorbid depression.	Bidirectional, saturable SP transport is supported in experimental in vitro BBB models; a CSU‐to‐BBB‐to‐psychiatric pathway has not been demonstrated.	[49, 50, 51, 52]
MRGPRX2‐mediated mast‐cell activation	Tissue‐expression, functional skin‐reactivity, circulating‐biomarker, and genetic studies support a role in CSU.	Links sensory neuropeptides to mast‐cell activation; relevance to CNS‐border mast cells or BBB regulation in CSU is unproven.	[53, 54, 55, 56, 57]
Stress, pruritus, and sleep pathways	Bidirectional stress effects and strong pruritus–sleep–mood associations are directly supported in CSU.	These established pathways can produce psychiatric symptoms without BBB dysfunction and must be modelled before invoking a neurovascular mechanism.	[8, 15, 58]

Abbreviations: CNS, central nervous system; CRP, C‐reactive protein; CSU, chronic spontaneous urticaria; MMP‐9, matrix metalloproteinase‐9; MRGPRX2, Mas‐related G protein‐coupled receptor X2; SP, substance P; TNF, tumour necrosis factor.

SP is of particular interest at the intersection of pruritus, stress, and neuroimmune signalling. Serum SP is increased in some CSU cohorts and has been associated with disease activity or comorbid depression [49, 50]. MRGPRX2‐mediated mast‐cell activation is supported by tissue expression, functional skin reactivity, circulating biomarkers, and genetic studies in CSU [53, 54, 55, 56, 57]. Experimental in vitro BBB models have reported bidirectional, saturable SP transport and suggested a carrier‐mediated process with pharmacological characteristics involving NK‐1 receptor recognition [51, 52]. These experimental observations do not show that circulating SP alters BBB function or causes psychiatric symptoms in CSU (Figure 2).

**FIGURE 2:**
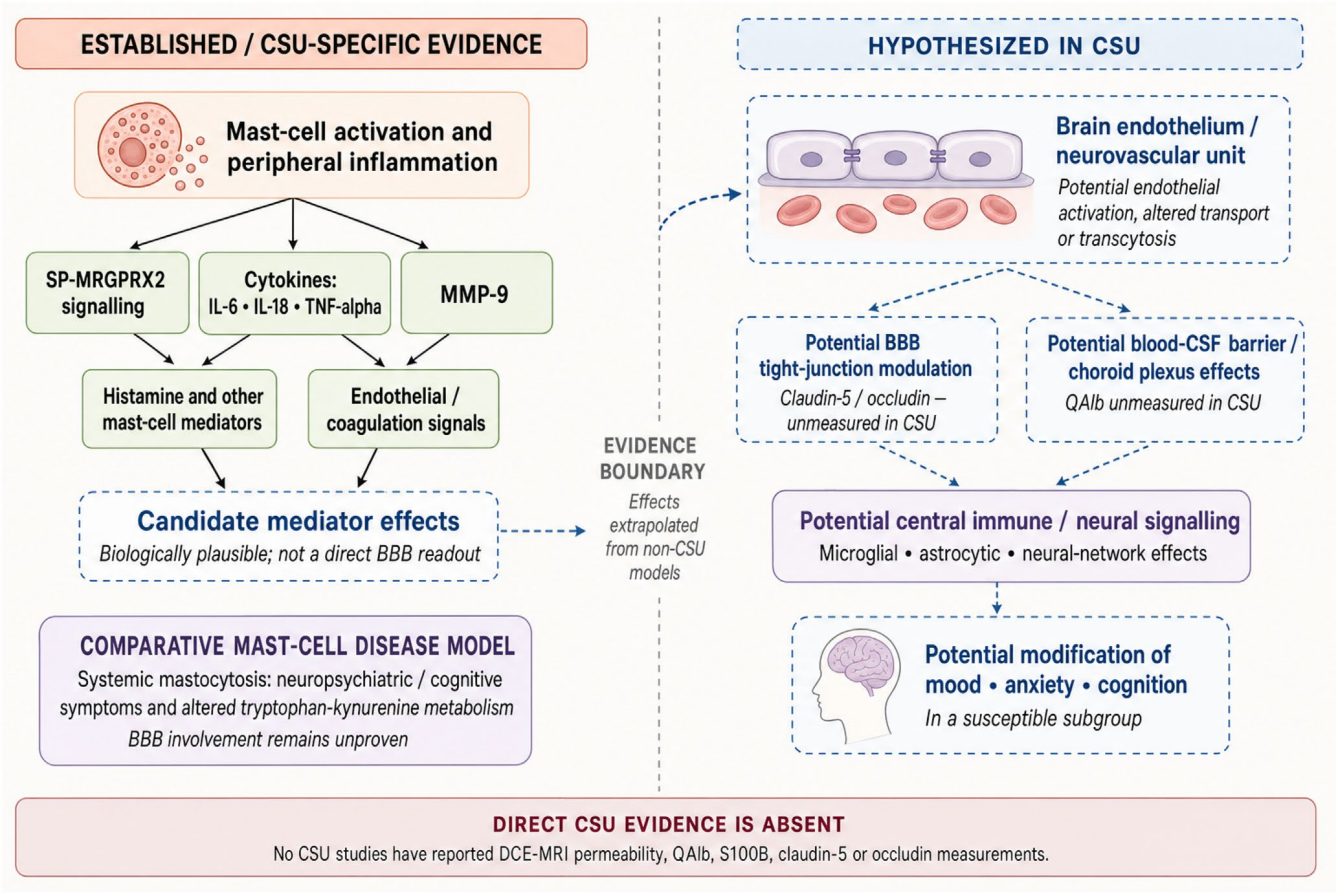
Candidate molecular pathways and the boundary between established and hypothesized evidence. CSU‐associated mast‐cell activation and peripheral inflammation generate mediators and vascular signals supported to varying degrees in CSU (solid boxes and arrows). Their putative effects on brain endothelium, BBB tight junctions, the blood–CSF barrier/choroid plexus, and central immune or neural signalling are extrapolated from non‐CSU inflammatory or experimental models and are shown with dashed elements. The CSF/serum albumin quotient (QAlb) is assigned specifically to the blood–CSF barrier/CSF‐dynamics compartment and is not treated as an interchangeable BBB permeability measure. Systemic mastocytosis provides a comparative model for mast‐cell‐related neuropsychiatric and cognitive manifestations but does not establish barrier involvement in CSU.

## 8 Systemic Mastocytosis as a Comparative Mast‐Cell Disease Model

Systemic mastocytosis is a relevant comparator because it combines chronic systemic mast‐cell activation with frequent neuropsychiatric and cognitive symptoms. A systematic review of 24 studies documented depression, anxiety or stress, cognitive impairment, and headache in mastocytosis [59]. In a large mastocytosis cohort, depressive symptoms were common, and a separate mechanistic study linked depression and perceived stress to altered tryptophan–kynurenine metabolism and higher indoleamine 2,3‐dioxygenase activity [60, 61]. Brain MRI and perfusion imaging have also reported morphological or functional abnormalities in patients with mastocytosis and psychocognitive complaints [62]. Although mastocytosis and CSU are biologically distinct, non‐skin symptoms such as fatigue and sleep disturbance are also common in CSU and are linked to active or uncontrolled disease [8].

These observations support the broader concept that sustained mast‐cell disease may be accompanied by CNS manifestations, but they do not establish barrier involvement. The cited mastocytosis studies did not directly assess regional BBB permeability or blood–CSF barrier function, and systemic mastocytosis is a clonal multisystem disorder with a mast‐cell burden and mediator profile that differ substantially from CSU. In addition, the imaging abnormalities were identified in patients selected for psychocognitive complaints and were not accompanied by permeability imaging or CSF barrier indices [59, 62]. Systemic mastocytosis should therefore be regarded as a comparative biological model, not as direct evidence for the CSU hypothesis.

## 9 Established Non‐BBB Pathways and Key Confounders

Pruritus, sleep disturbance, fatigue, and psychosocial burden are established contributors rather than merely confounding factors and may explain much of the psychiatric association in CSU. In the adolescent study noted above, the associations of CSU with anxiety and depression were no longer significant after accounting for itching and sleep disturbance [15]. Poor sleep is associated with worse disease control, greater pruritus, impaired quality of life, and higher anxiety and depression risk, whereas effective CSU treatment can improve sleep [5, 6, 7, 8]. These pathways should remain the primary clinical focus even when neurovascular mechanisms are investigated.

Medication exposure and comorbidity also complicate causal interpretation. Although first‐generation H1‐antihistamines are not recommended for routine CSU treatment, their central H1‐receptor occupancy can cause sedation, psychomotor impairment, and cognitive slowing. Therefore, second‐generation antihistamines with lower CNS penetration are preferred [1, 63, 64, 65]. Cardiometabolic disease, smoking, systemic inflammation, psychotropic medication, and acute illness can influence both psychiatric symptoms and barrier‐related measures [25, 26]. Psychological stress may exacerbate CSU through neuroendocrine, autonomic, behavioural, and perceptual pathways, creating bidirectional amplification without requiring BBB leakage [15, 18, 19, 58]. Clinical observations further suggest that improvement in CSU may accompany improvement in psychological symptoms, and vice versa. Perceived stress, anxiety, and depression scores improved during 24 weeks of omalizumab treatment [66], while a recent case report described sustained improvement in refractory CSU following cognitive behavioural therapy and escitalopram, with relapse after treatment withdrawal or major stressful events and renewed disease control after psychiatric treatment was resumed [22]. Together, these observations are consistent with a bidirectional relationship between CSU and psychological factors, although neither study establishes causality or provides evidence of BBB normalization.

## 10 Discussion

This review brings together established epidemiological and symptom‐related pathways with indirect neurovascular evidence to propose a testable, non‐exclusive hypothesis. Pruritus, sleep disturbance, fatigue, and psychosocial burden remain the best‐supported contributors to psychiatric symptoms in CSU [5, 6, 7, 8, 15]. The BBB/neurovascular framework may modify disease in a susceptible subgroup, but the features defining that subgroup remain unknown. It is not a confirmed component of CSU pathophysiology. This perspective does not diminish the clinical importance of psychiatric comorbidity; rather, it places the proposed biological pathway within the broader, better‐supported clinical framework and specifies the observations needed to determine whether it contributes independently.

At present, the framework is strictly hypothesis‐generating and does not justify changes to clinical treatment. It separates direct CSU observations (psychiatric burden, peripheral endothelial and coagulation signals, SP–MRGPRX2 biology, and non‐permeability brain imaging) from findings extrapolated from psychiatric, inflammatory, experimental, or systemic mast‐cell disease. Its value will depend on whether direct neurovascular measurements add explanatory or predictive information beyond the established pruritus–sleep–mood pathway.

Investigating BBB/neurovascular dysfunction may also have implications for CSU endotyping. If reproducible neurovascular abnormalities are linked to a distinct, disease‐relevant mechanism rather than nonspecific symptom burden or comorbidity, they could help identify previously unrecognized CSU endotypes or refine existing ones. Such endotypes might benefit more from particular therapies, potentially allowing better matching of patients to treatment. This possibility should be tested separately for cutaneous disease control and neuropsychiatric outcomes and would not require effective treatment to act directly on the BBB. These are potential future applications, contingent on mechanistic confirmation and prospective validation of treatment‐response differences, rather than current therapeutic recommendations.

A subgroup formulation also clarifies why psychiatric manifestations are not universal among patients with CSU. Even if a neurovascular effect is eventually validated, it would be neither necessary nor sufficient for psychiatric illness. Clinical expressions would likely depend on CSU endotype and activity, cumulative pruritus and sleep deprivation, stress susceptibility, medication exposure, cardiometabolic comorbidity, and individual resilience. Transient or regionally restricted changes may also be insufficient, on their own, to produce clinically significant psychiatric manifestations [9, 10, 11, 12, 13, 14, 15, 25, 26, 46, 47, 48, 49, 50, 51, 52, 53, 54, 55, 56, 57, 58]. This multiple‐hit model is more consistent with the heterogeneity of CSU and brain‐barrier findings than a deterministic CSU–BBB–psychiatric sequence.

By integrating CSU‐specific psychiatric, vascular, neuroimmune, and neuroimaging evidence, this review distinguishes direct findings from comparative and mechanistic extrapolation and places the proposed neurovascular hypothesis alongside established clinical pathways and relevant confounders. Several limitations should be emphasized. Direct evidence of altered regional BBB permeability, blood–CSF barrier function, or neurovascular‐unit pathology in CSU is absent, so the proposed links remain inferential. Peripheral endothelial markers such as MMP‐9 are nonspecific and cannot serve as surrogate evidence of BBB dysfunction. S100B, QAlb, DCE‐MRI, and tight‐junction measurements interrogate different biological compartments or processes and are not interchangeable. Some CSU neuroimaging reports may include overlapping cohorts, and none assessed permeability or neuroinflammation. Systemic mastocytosis provides a comparative mast‐cell–CNS model but differs fundamentally from CSU. Finally, observational associations remain vulnerable to confounding and reverse causation from pruritus, sleep disturbance, medication exposure, cardiometabolic disease, and psychiatric illness itself.

## 11 Major Open Questions and How to Answer Them

At present, no direct human evidence demonstrates altered regional BBB permeability or blood–CSF barrier function in CSU. The key question is therefore whether neurovascular abnormalities occur in a subset of patients and, if so, whether they provide explanatory value beyond pruritus, sleep disturbance, fatigue, psychosocial burden, and treatment‐related effects. Table 2 outlines a staged approach to addressing these questions.

**TABLE 2:** Priority research questions and suggested study designs.

Priority question	Suggested design	Key measures	Rationale	Supporting evidence (ref #)
Are regional BBB permeability or blood–CSF barrier abnormalities present in CSU?	Prospective case–control study stratified by activity, endotype, and psychiatric status.	UAS7/UCT; sleep and psychiatric phenotyping; DCE‐MRI for regional BBB permeability; QAlb/CSF total protein for blood–CSF barrier function or CSF dynamics where ethically justified.	Determines whether any barrier abnormality exists and avoids conflating distinct compartments.	[25, 26, 27, 28, 29]
Do neurovascular measures add information beyond pruritus and sleep?	Multivariable longitudinal cohort with prespecified mediator and confounder models.	Pruritus, sleep, fatigue, psychosocial burden, antihistamine/psychotropic exposure, inflammatory markers, and neurovascular measures.	Tests incremental explanatory value beyond established pathways.	[8, 15]
Do candidate measures change with CSU control?	Pre–post cohort during guideline‐based treatment escalation, with repeated measures.	Disease control, sleep and mental‐health outcomes, selected endothelial/neurovascular measures, and optional neurocognitive testing.	Tests temporal coupling without assuming causality.	[1, 6, 38, 66]
Does SP–MRGPRX2 signalling mark a high‐risk phenotype?	Mechanistic cohort with biomarker subtyping and longitudinal analysis.	SP, MRGPRX2, inflammatory markers, pruritus/sleep measures, and standardized psychiatric phenotyping.	Determines whether neuropeptide–mast‐cell activation relates to psychiatric symptoms independently of symptom burden.	[49, 50, 53, 54, 55, 56, 57]
Can neurovascular profiles refine endotyping and predict differential treatment benefit?	Prospective mechanistic cohorts followed by biomarker‐stratified randomized comparative trials.	Baseline neurovascular profile; existing endotypes; disease severity; separate cutaneous and neuropsychiatric outcomes; prespecified treatment‐by‐biomarker interactions.	Distinguishes mechanistic endotyping and predictive utility from a nonspecific association with severity.	[1, 46, 47]^a^

Abbreviations: BMI, body mass index; DCE‐MRI, dynamic contrast‐enhanced magnetic resonance imaging; QAlb, CSF/serum albumin quotient; SP, substance P; UAS7, Urticaria Activity Score over 7 days; UCT, Urticaria Control Test.

^a^ Background on CSU endotyping and treatment; neurovascular endotypes and their treatment‐predictive value remain unproven.

Future studies should combine complementary measures rather than group them broadly as “BBB biomarkers.” DCE‐MRI can estimate regional BBB permeability, whereas QAlb and CSF total protein primarily reflect blood–CSF barrier function and CSF dynamics, and circulating S100B is a nonspecific marker influenced by astroglial and extracranial sources [25, 26, 27, 28, 29]. Among these approaches, DCE‐MRI is the most direct proposed noninvasive method for testing regional BBB permeability in CSU, provided that acquisition and analysis are standardized and practical considerations such as cost and gadolinium exposure are addressed [27, 28, 29].

Initial studies should stratify participants by disease activity and control (UAS7/UCT), mechanistic endotype where feasible, and systemic inflammatory phenotype. Standardized assessments of depression, anxiety, sleep, and fatigue should be included, together with assessment of suicidal ideation when clinically indicated and relevant potential confounders such as BMI, smoking, cardiometabolic disease, psychotropic medication, and antihistamine exposure [25, 26, 46, 47]. Longitudinal assessment across active disease, treatment response, and remission will be essential to establish temporality and determine whether neurovascular measures add information beyond established symptom‐related pathways. If a reproducible neurovascular profile emerges, subsequent biomarker‐stratified comparative trials should test whether it predicts differential treatment benefit beyond existing endotypes and baseline disease severity, using prespecified treatment‐by‐biomarker interactions and separate cutaneous and neuropsychiatric outcomes. Conversely, a null result across adequately powered, compartment‐appropriate assessments after adjustment for symptom burden and key confounders would argue against a generalized CSU–BBB mechanism and restrict any residual hypothesis to a more narrowly defined subgroup.

## 12 Conclusions and Perspectives

CSU is associated with a substantial burden of anxiety, depression, sleep disturbance, fatigue, and functional impairment, while recent incident‐outcome data suggest that the risk of new psychiatric disease is narrower than the prevalent comorbidity burden would imply [9, 10, 11, 12, 13, 14, 15, 20]. Persistent pruritus, impaired sleep, and psychosocial effects account for an important proportion of the observed association and remain the principal clinical targets. The BBB/neurovascular unit represents a plausible but unproven additional interface that may modify psychiatric risk in a subgroup. Direct, compartment‐appropriate measurements in endotype‐stratified longitudinal cohorts are required to establish mechanistic relevance; if validated, these findings could reveal or refine CSU endotypes and support future treatment stratification.

## Author Contributions

Gerasimos N. Konstantinou and George N. Konstantinou contributed equally to the conceptualization of the work, literature review and synthesis, interpretation of the evidence, drafting of the manuscript, and critical revision for important intellectual content. Jerry J. Warsh, Daniel M. Blumberger, and Stefan Kloiber contributed to the interpretation of the evidence and critically reviewed and revised the manuscript for important intellectual content. All authors read and approved the final manuscript and agree to be accountable for all aspects of the work.

## Funding

This research received no specific grant from funding agencies in the public, commercial, or not‐for‐profit sectors.

## Conflicts of Interest

The authors declare no conflicts of interest.

## Data Availability

Data sharing not applicable to this article as no datasets were generated or analysed during the current study.
